# A Challenge toward Novel Quaternary Sulfides SrLnCuS_3_ (Ln = La, Nd, Tm): Unraveling Synthetic Pathways, Structures and Properties

**DOI:** 10.3390/ijms232012438

**Published:** 2022-10-18

**Authors:** Anna V. Ruseikina, Maxim V. Grigoriev, Leonid A. Solovyov, Vladimir A. Chernyshev, Aleksandr S. Aleksandrovsky, Alexander S. Krylov, Svetlana N. Krylova, Nikolai P. Shestakov, Dmitriy A. Velikanov, Alexander A. Garmonov, Alexey V. Matigorov, Marcel A. Eberle, Thomas Schleid, Damir A. Safin

**Affiliations:** 1 Laboratory of Theory and Optimization of Chemical and Technological Processes, University of Tyumen, 625003 Tyumen, Russia; 2 Federal Research Center KSC SB RAS, Institute of Chemistry and Chemical Technology, 660036 Krasnoyarsk, Russia; 3 Institute of Natural Sciences and Mathematics, Ural Federal University named after the First President of Russia B.N. Yeltsin, Mira Str. 19, 620002 Ekaterinburg, Russia; 4 Kirensky Institute of Physics, Federal Research Center KSC SB RAS, 660036 Krasnoyarsk, Russia; 5 Department of Photonics and Laser Technology, Siberian Federal University, 660079 Krasnoyarsk, Russia; 6 Institute of Physics and Technology, University of Tyumen, Volodarskogo Str. 6, 625003 Tyumen, Russia; 7 Institute of Inorganic Chemistry, University of Stuttgart, D-70569 Stuttgart, Germany; 8 Scientific and Educational and Innovation Center for Chemical and Pharmaceutical Technologies, Ural Federal University named after the First President of Russia B.N. Yeltsin, Mira Str. 19, 620002 Ekaterinburg, Russia; 9 «Advanced Materials for Industry and Biomedicine» Laboratory, Kurgan State University, Sovetskaya Str. 63/4, 640020 Kurgan, Russia; 10 University of Tyumen, Volodarskogo Str. 6, 625003 Tyumen, Russia

**Keywords:** inorganic materials, quaternary sulfide, synthesis, crystal structure, ab initio calculations, magnetic measurements, spectroscopy

## Abstract

We report on the novel heterometallic quaternary sulfides SrLnCuS_3_ (Ln = La, Nd, Tm), obtained as both single crystals and powdered samples. The structures of both the single crystal and powdered samples of SrLaCuS_3_ and SrNdCuS_3_ belong to the orthorhombic space group *Pnma* but are of different structural types, while both samples of SrTmCuS_3_ crystallize in the orthorhombic space group *Cmcm* with the structural type KZrCuS_3_. Three-dimensional crystal structures of SrLaCuS_3_ and SrNdCuS_3_ are formed from the (Sr/Ln)S_7_ capped trigonal prisms and CuS_4_ tetrahedra. In SrLaCuS_3_, alternating 2D layers are stacked, while the main backbone of the structure of SrNdCuS_3_ is a polymeric 3D framework [(Sr/Ln)S_7_]_n_, strengthened by 1D polymeric chains (CuS_4_)_n_ with 1D channels, filled by the other Sr^2+^/Ln^3+^ cations, which, in turn, form 1D dimeric ribbons. A 3D crystal structure of SrTmCuS_3_ is constructed from the SrS_6_ trigonal prisms, TmS_6_ octahedra and CuS_4_ tetrahedra. The latter two polyhedra are packed together into 2D layers, which are separated by 1D chains (SrS_6_)_n_ and 1D free channels. In both crystal structures of SrLaCuS_3_ obtained in this work, the crystallographic positions of strontium and lanthanum were partially mixed, while only in the structure of SrNdCuS_3_, solved from the powder X-ray diffraction data, were the crystallographic positions of strontium and neodymium partially mixed. Band gaps of SrLnCuS_3_ (Ln = La, Nd, Tm) were found to be 1.86, 1.94 and 2.57 eV, respectively. Both SrNdCuS_3_ and SrTmCuS_3_ were found to be paramagnetic at 20–300 K, with the experimental magnetic characteristics being in good agreement with the corresponding calculated parameters.

## 1. Introduction

Layered chalcogenides containing *d*-metals are of particular interest due to their valuable properties, as well as being used as superconductors [1], magnetic [2,3] and thermoelectric materials [4], materials for infrared and nonlinear optics [5,6,7] and catalysts [8]. The quaternary chalcogenides ABCX_3_ (A is an alkali or alkaline earth metal, Eu; B is a *d*- or *f*-element; C is a *d*-element; X is a chalcogenide) produce layered and/or channel structures, crystallizing in various structural types [9,10,11,12,13,14,15,16,17,18,19,20,21,22,23]. An overwhelming majority of the crystal structures of ABCX_3_ exhibit 2D anionic layers [BCX_3_]^n−^ with trapped A^n+^ cations. Both the nature and charge of A^n+^ affect the lattice distortion, electronic structure, phonon dispersion, electrical properties, and heat transfer properties in the crystal structure [24]. ABCX_3_ compounds exhibit very low lattice thermal conductivity [25,26,27], promising thermoelectric characteristics [24,28] and high photovoltaic efficiency [27]. Thus, thermodynamically stable quaternary compounds are potential high-performance thermoelectrics, thermal barrier coatings, and thermal data-storage devices [26,29,30,31,32]. Furthermore, semiconductor, magnetic, optical, and thermodynamic properties have also been described for these compounds [4,9,10,11,12,13,14,15,16,17,18,19,20,23,24,33,34,35,36,37,38].

Of the ABCX_3_ compounds, the quaternary chalcogenides ALnCuX_3_ are of particular interest, since transition and rare earth elements exhibit rich crystal chemistry and specific spectroscopic properties. According to quantum mechanical calculations, the band gap of SrLnCuS_3_ is 1.00–1.51 eV [26], indicating that these compounds are promising materials for solar cells with an efficiency of about 20% [39,40,41,42]. However, the lack of experimental data on the band gap of SrLnCuS_3_ limits their practical utilization.

Polycrystalline samples of SrLnCuS_3_ were obtained by the melting of a stoichiometric ratio of sulfides SrS, Ln_2_S_3_ and Cu_2_S, followed by annealing at 970–1170 K for 2–3 (!) months [22,34,35,36,43,44,45]. Single-crystal samples were synthesized from elemental Sr, Cu and Ln (Ln = Nd, Y, Sc) using a halide flux at 1070 K for 8 days [46,47]. Recently, we have reported on the synthesis of EuLnCuS_3_ by the sulfidation of a mixture of oxides obtained by thermolysis of co-crystallized copper nitrate and rare-earth nitrates for 25 h [48]. Thus, using this approach would, likely, significantly reduce the synthesis time towards SrLnCuS_3_. Notably, strontium oxide is more active than europium oxide with respect to the material of the reactor used for synthesis due to the presence of an alkaline earth element. Therefore, it is suggested that the initial synthesis temperatures to produce SrLnCuS_3_ should be lower than those of EuLnCuS_3_.

In the literature, the crystal structures of some SrLnCuS_3_ have already been reported [21,22,34,35,36,43,44,45,46,47]. For SrLnCuS_3_, two types of orthorhombic crystal structures with *Pnma* symmetry and one type with *Cmcm* symmetry were revealed [22]. Sulfides SrLnCuS_3_ (Ln = La–Nd) crystallize in the BaLaCuS_3_ structural type, and the crystallographic positions of the Sr^2+^ and Ln^3+^ ions are partially mixed by 21% in SrLaCuS_3_ [22,43], by 16% in SrCeCuS_3_ [44] and by 11% in SrPrCuS_3_ [22,43], while in SrNdCuS_3_ the Sr^2+^ and Ln^3+^ cations occupy two different crystallographic positions [46]. It should also be noted that, for SrNdCuS_3_, the second modification of the structural type Eu_2_CuS_3_ was obtained [46], while for SrCeCuS_3_, a high-temperature polymorphic modification of the structural type Ba_2_MnS_3_ with a partially mixed crystallographic positions of the Sr^2+^ and Ce^3+^ ions was also revealed [44].

The isostructural quaternary sulfides EuLnCuS_3_ [37,49] and selenides EuLnCuSe_3_ [23] and SrLnCuSe_3_ [50,51] always start to crystallize in the structural type Ba_2_MnS_3_. Due to the similar ionic radii of Eu^2+^ and Sr^2+^ (1.17 and 1.18 Å, respectively [52]), the structural types of EuLnCuS_3_ and SrLnCuS_3_ should also be similar. Thus, it can tentatively be assumed that SrLaCuS_3_ will also crystallize in the structural type Ba_2_MnS_3_. Sulfides SrLnCuS_3_ (Ln = Sm [22], Gd [22], Ho [35], Y [47]) belong to the structural type Eu_2_CuS_3_, while SrLnCuS_3_ (Ln = Er [45], Yb [45], Lu [22], Sc [36]) are of the structural type KZrCuS_3_. In all the listed compounds, the Eu^2+^ and Ln^3+^ ions occupy two crystallographically independent positions. The change of space group from *Pnma* to *Cmcm* occurs between SrYCuS_3_ and SrErCuS_3_ [23]. SrLnCuS_3_ melt incongruently at 1429–1712 K [34,43]. SrLaCuS_3_ and SrPrCuS_3_ are optically transparent in the IR range from 1800 to 3600 cm^−1^ [43]. To the best of our knowledge, other physical properties of sulfides SrLnCuS_3_ have not been reported so far.

With all this in mind, in this work we have focused on the synthesis of SrLnCuS_3_ (Ln = La, Nd, Tm), as well as studying their crystal structures, magnetic and optical properties.

## 2. Results and Discussion

The heterometallic quaternary sulfides SrLnCuS_3_ (Ln = La, Nd, Tm) were obtained both in the form of single crystals as well as powdered samples. The former synthetic procedure requires heating a stoichiometric ratio of the elements Sr, Cu, Ln and S in the presence of CsBr as a flux for 8 days at 1070 K, while the latter synthetic approach significantly decreases the reaction time in comparison to both a single-crystal method and the sulfide-melting method [22,35,43,44,45,47].

To date, for the quaternary sulfides, only four crystal structures of SrLnCuS_3_ (Ln = Nd, Y, Sc), solved from the single-crystal X-ray diffraction data, have been known so far (Table 1) [46,47], while twelve crystal structures of a series of SrLnCuS_3_ (Ln = La, Ce, Pr, Nd, Sm, Gd, Ho, Er, Tm, Yb, Lu, Sc), solved from the powder X-ray diffraction data, have been reported (Table 2) [22,36,44,45].

In the present work, we report for the first time the crystal structures of SrLaCuS_3_ and SrTmCuS_3_ (Table 1), solved from the single-crystal X-ray diffraction data, as well as the crystal structure of SrLnCuS_3_ (Ln = La, Nd, Tm), solved from the powder X-ray diffraction data (Table 2). Notably, the same synthetic approach for the formation of single crystals yields one structural type, Ba_2_MnS_3_, for SrLaCuS_3_, and two structural types, BaLaCuS_3_ and Eu_2_CuS_3_, for SrNdCuS_3_ of the orthorhombic space group *Pnma*, while for SrTmCuS_3_, the structural type KZrCuS_3_ of the orthorhombic space group *Cmcm* was established (Table 1). However, different synthetic approaches toward powdered samples yield two structural types, BaLaCuS_3_ and Ba_2_MnS_3_, for SrLaCuS_3_, one structural type, BaLaCuS_3_, for SrNdCuS_3_ of the same orthorhombic space group *Pnma*, and one structural type, KZrCuS_3_, of the orthorhombic space group *Cmcm* for SrTmCuS_3_ (Table 2). Interestingly, while the *b* axis in the structures of SrLaCuS_3_ and SrNdCuS_3_ is very similar and varies from 4.0072(3) Å to 4.11053(6) Å, the *a* and *c* axes differ significantly ranging from 8.1682(6) Å to 16.0394(11) Å (Table 1 and Table 2). Furthermore, the cell volume is almost the same in the structures of SrLaCuS_3_, regardless of the structural type, and of 533.85(7)–535.97(1) Å^3^, but differs in the structures of SrNdCuS_3_. Particularly, the cell volume is 518.63(2)–519.14(1) Å^3^ in the structural type BaLaCuS_3_, and is remarkably larger in the structural type Eu_2_CuS_3_ (545.96(3) Å^3^). The cell volume in the structures of SrLaCuS_3_ is smaller and of 510.50(6)–511.34(3) Å^3^.

It should also be noted that in both crystal structures of SrLaCuS_3_ obtained in this work, the crystallographic positions of strontium and lanthanum were partially mixed by about 50% and 45%, respectively, while only in the structure of SrNdCuS_3_, solved from the powder X-ray diffraction data, the crystallographic positions of strontium and neodymium were partially mixed by about 11% (Table 3). In both structures of SrTmCuS_3_, each atom fully occupies its own crystallographical position (Table 3).

**Figure 1:**
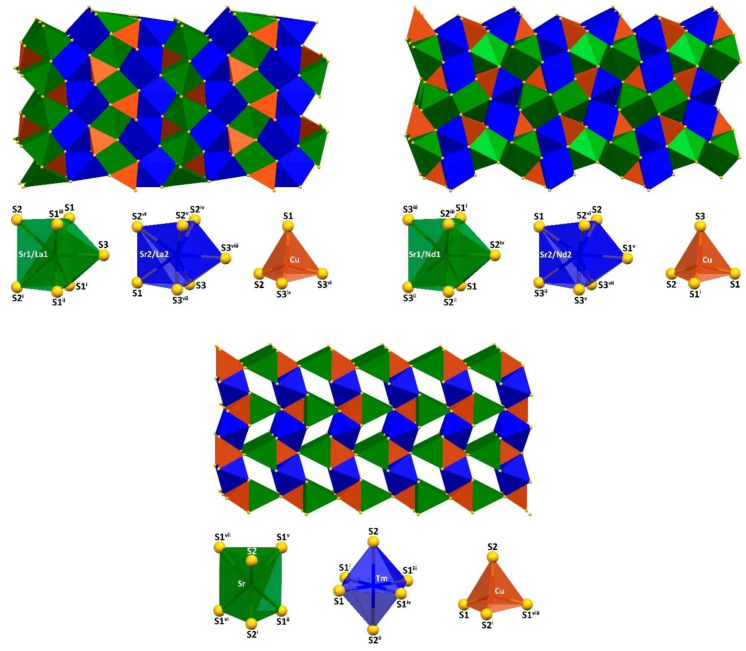
View of the crystal structures of SrLnCuS_3_ (Ln = La, top-left; Nd, top-right, Tm, bottom) together with the coordination polyhedra formed by the metal ions. Color code: green polyhedron = (Sr1/La1)S_7_, (Sr1/Nd1)S_7_ and SrS_6_; blue polyhedron = (Sr2/La2)S_7_, (Sr2/Nd2)S_7_ and TmS_6_; burnt orange polyhedron = CuS_4_. Symmetry codes for SrLaCuS_3_: (i) *x*, –1 + *y*, *z*; (ii) 1/2 + *x*, −1/2 − *y*, 1/2 − *z*; (iii) 1/2 + *x*, 1/2 − *y*, 1/2 − *z*; (iv) 1/2 − *x*, −*y*, −1/2 + *z*; (v) 1/2 − *x*, 1 − *y*, −1/2 + *z*; (vi) −1/2 + *x*, 1/2 − *y*, 1/2 − *z*; (vii) *x*, 1 + *y*, *z*; (viii) 1 − *x*, 1/2 + *y*, −*z*; (ix) −1/2 + *x*, 1/2 − *y*, 1/2 − *z*. Symmetry codes for SrNdCuS_3_: (i) *x*, −1 + *y*, *z*; (ii) 1/2 − *x*, −*y*, 1/2 + *z*; (iii) 1/2 − *x*, −1 − *y*, 3/2 − *z*; (iv) −1/2 + *x*, −1/2 − *y*, 3/2 − *z*; (v) 1/2 + *x*, 1/2 − *y*, 3/2 − *z*; (vi) *x*, 1 + *y*, *z*; (vii) 1/2 + *x*, −1/2 − *y*, 3/2 − *z*. Symmetry codes for SrTmCuS_3_: (i) −*x*, −*y*, −*z*; (ii) −*x*, −*y*, −1/2 + *z*; (iii) 1 + *x*, *y*, *z*; (iv) 1 + *x*, −*y*, −*z*; (v) −1/2 − *x*, 1/2 + *y*, 1/2 − *z*; (vi) −1/2 + *x*, 1/2 + *y*, *z*; (vii) −3/2 − *x*, 1/2 + *y*, 1/2 − *z*; (viii) −1 − *x*, *y*, 1/2 − *z*.

A 3D crystal structure of both SrLaCuS_3_ and SrNdCuS_3_ was constructed from the (Sr1/Ln1)S_7_- and (Sr2/Ln2)S_7_-capped trigonal prisms as well as CuS_4_ tetrahedra (Figure 1). However, different structural types of these compounds are clearly reflected in the packing of coordination polyhedra. Particularly, in the structure of SrLaCuS_3_, the (Sr1/La1)S_7_-and (Sr2/La2)S_7_-capped trigonal prisms each form alternating 2D layers, of which the (Sr1/La1)S_7_-based layers are further strengthened by 1D polymeric chains (CuS_4_)_n_ (Figure 1). The main backbone of the structure of SrNdCuS_3_ is a polymeric 3D framework [(Sr1/Ln1)S_7_]_n_, further strengthened by 1D polymeric chains (CuS_4_)_n_, with 1D channels along the *b* axis, filled by the Sr2^2+^/Ln2^3+^ cations, which, in turn, form 1D dimeric ribbons along the *b* axis (Figure 1). A 3D crystal structure of SrTmCuS_3_ differs significantly from the La- and Nd-based derivatives and is constructed from the SrS_6_ trigonal prisms and TmS_6_ octahedra as well as CuS_4_ tetrahedra (Figure 1). The latter two polyhedra are packed together into 2D layers, which are separated by 1D chains (SrS_6_)_n_ and 1D free channels along the *a* axis (Figure 1).

In the discussed structures of SrLaCuS_3_, the Sr/La–S bond lengths are 2.910(1)–3.09999(17) Å, while the Sr/Nd–S bonds in the structures of SrNdCuS_3_ vary in a broader range from 2.843(1) Å to 3.136(2) Å (Table 4). The Sr–S bond lengths in the structures of SrTmCuS_3_ are similar to those in the La- and Nd-based derivatives, and of 2.966(3)–3.093(2) Å, while the Tm–S bonds are shorter and of 2.703(2)–2.719(2) Å (Table 4). The Cu–S distances within the coordination tetrahedron in all the reported structures vary from 2.325(2) Å to 2.388(3) Å (Table 4).

**Figure 2:**
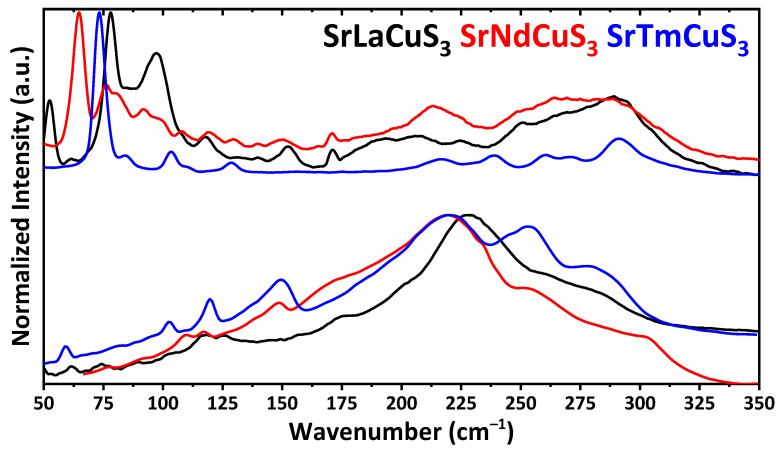
The IR (bottom) and Raman (top) spectra of SrLnCuS_3_ (Ln = Sr, Nd, Tm).

The IR and Raman spectra of SrLnCuS_3_ (Ln = La, Nd, Tm) each contain bands exclusively up to about 350 cm^−1^ (Figure 2), thus the discussed compounds are both IR and Raman transparent, at least in the region of 350–4000 cm^−1^. The most intense bands in the IR and Raman spectra were observed at about 220–230 and 65–80 cm^−1^, respectively.

**Figure 3:**
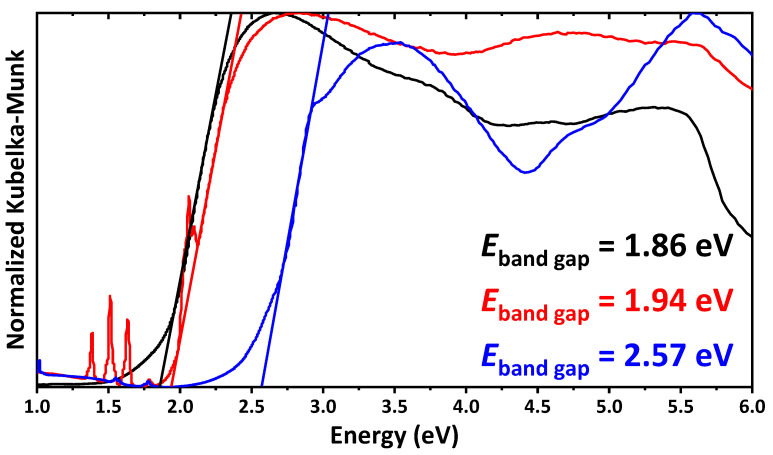
The normalized Kubelka–Munk spectra of SrLaCuS_3_ (black), SrNdCuS_3_ (red) and SrTmCuS_3_ (blue). Sharp bands at about 1.30–1.80 and 2.06 in the spectrum of SrNdCuS_3_ correspond to different transitions of Nd^3+^ [53].

For SrLnCuS_3_ (Ln = La, Nd, Tm), the experimental band gaps were obtained from the Kubelka–Munk function, and are of 1.86, 1.94 and 2.57 eV, respectively (Figure 3). Notably, the band gap values for SrLaCuS_3_ and SrNdCuS_3_ are similar, which might tentatively be explained by the same orthorhombic space group *Pnma* in the crystal structures of these sulfides, while the crystal structure of the discussed SrTmCuS_3_ exhibits the orthorhombic space group *Cmcm*.

**Figure 4:**
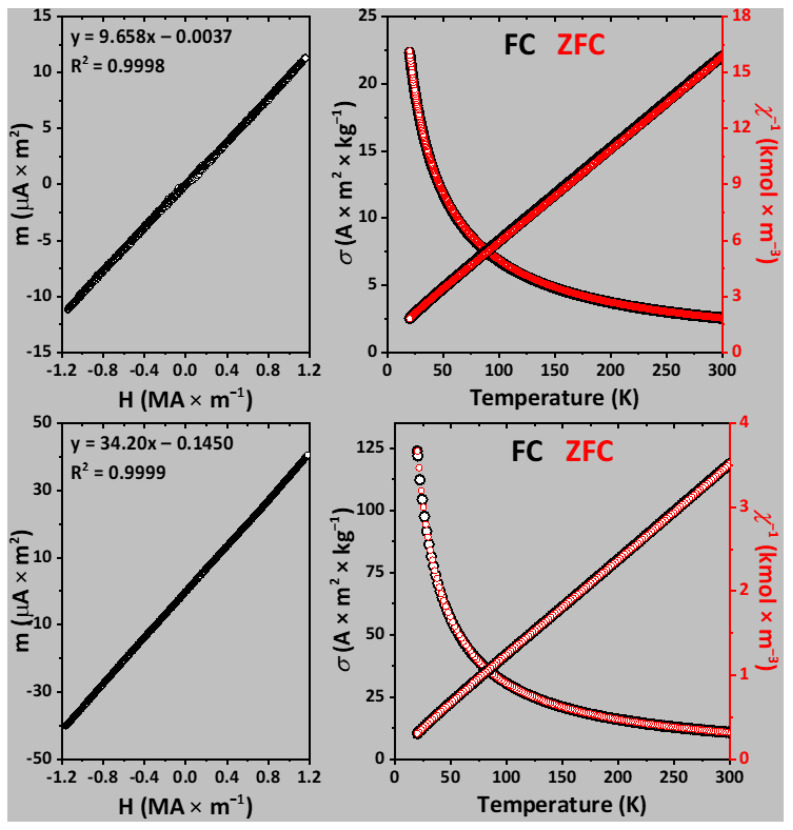
Field-dependent magnetic moments at 296 K (**left**), and temperature-dependent specific magnetization and reciprocal magnetic susceptibility (**right**) of SrNdCuS_3_ (**top**) and SrTmCuS_3_ (**bottom**) at 200 Oe. The temperature-dependent measurements were performed in the zero-field cooled (ZFC) and nonzero-field cooled (FC) modes.

The field-dependent magnetic moment of both SrNdCuS_3_ and SrTmCuS_3_ at 296 K is linear, which is characteristic for a paramagnetic compound (Figure 4). From this dependence, the effective magnetic moment was calculated as 3.611 and 7.378 μ_B_ for the Nd- and Tm-based sulfides, respectively (Table 5). The temperature-dependent reciprocal magnetic susceptibility at 20–300 K is well described by the Curie–Weiss law and is the same in both the zero-field cooled (ZFC) and nonzero-field cooled (FC) modes (Figure 4). As such, the *C*, *μ* and *θ* values were calculated for both compounds at 20–300 K (Table 5). Experimental magnetic characteristics for SrNdCuS_3_ and SrTmCuS_3_ are in good agreement with the corresponding calculated parameters, obtained in the model of free ions Nd^3+^ and Tm^3+^, respectively (Table 5).

## 3. Materials and Methods

### 3.1. Materials

Sr (99.2%), La (99.9%), Tm (99.9%) and CsBr (99.9%) were purchased from ChemPur (Karlsruhe, Germany). Ln_2_O_3_ (Ln = La, Nd, Tm; 99.95%) were purchased from the Uralredmet manufacture (Verkhnyaya Pyshma, Russia). Cu (99.9%) was obtained from SZB Tsvetmet, OJSC (Saint Petersburg, Russia), while Cu (99.8%) was purchased from Merck (Darmstadt, Germany). S (99.5%) was purchased from Alfa Aesar (Karlsruhe, Germany). Argon (99.998%) was purchased from Kislorod-Servis (Yekaterinburg, Russia). Concentrated nitric acid (extra-pure grade, 18-4 all-Union State Standard 11125-84) was purchased from Chemreaktivsnab, CJSC (Ufa, Russia). NH_4_SCN (98%) was obtained from Vekton Ltd. (Saint Petersburg, Russia). SrCO_3_ (99.8%) was purchased from VitaReaktiv LLC (VitaHim Group, Dzerzhinsk, Russia).

### 3.2. Methods

The single-crystal X-ray diffraction data for SrLaCuS_3_ and SrTmCuS_3_ were collected at room temperature with a Bruker–Nonius κ-CCD diffractometer (Mo-Kα radiation, graphite monochromator) equipped with a CCD detector. The collected intensity data and the numerical correction of the absorption for the measured crystals were processed using the DENZO [54] and HABITUS [55] programs, respectively. The crystal structures were solved and refined using the SHELX-2013 software package [56,57].

**Figure 5:**
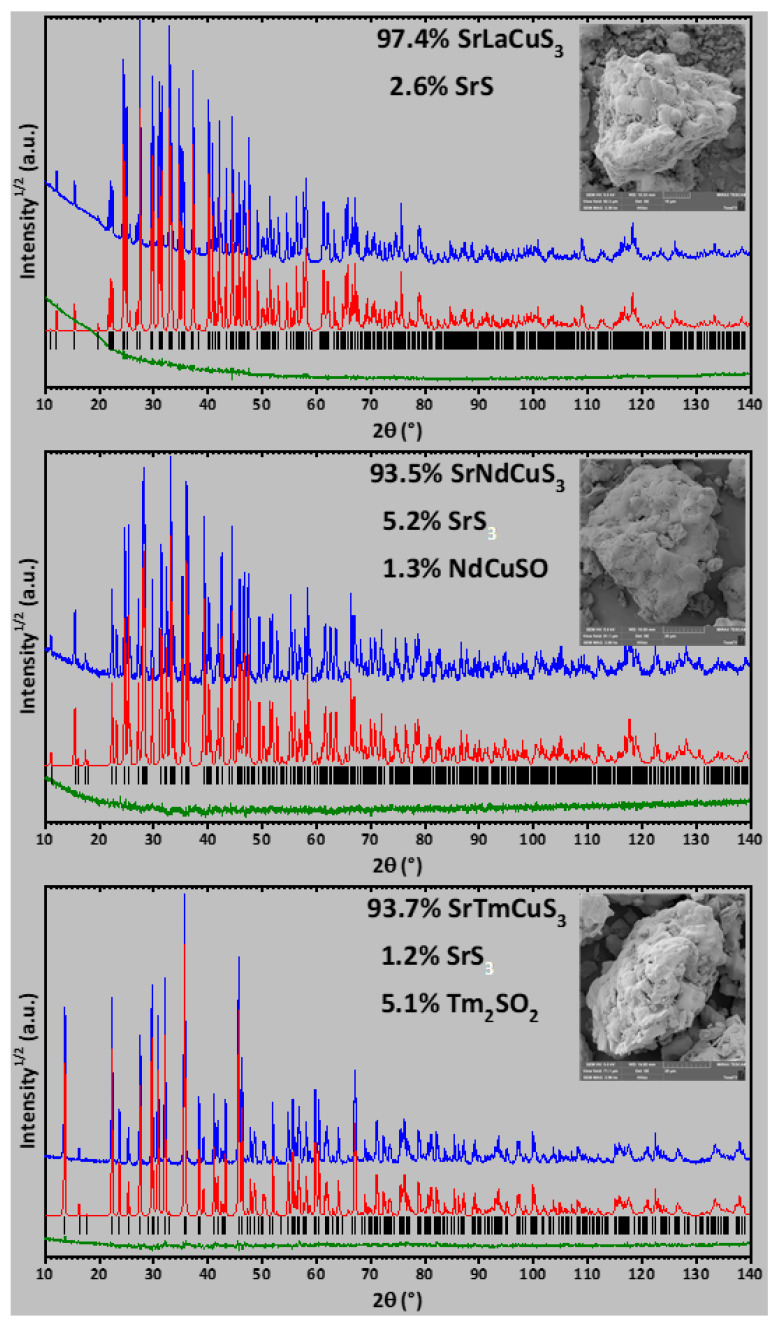
Observed (blue), calculated (red) and difference (green) X-ray powder diffraction patterns for SrLnCuS_3_ after crystal structure refinement. Insets show the SEM images of SrLnCuS_3_.

The powder X-ray diffraction data for SrLnCuS_3_ (Ln = La, Nd, Tm; Figure 5) were collected at room temperature with a ДPOH 7 (Burevestnik, Saint Petersburg, Russia) powder diffractometer (Cu-Kα radiation, graphite monochromator). The step size of 2θ was 0.02°, and the counting time was 35 s per step. The lattice parameters were determined using the ITO program [58] and the crystal structures were refined by the derivative difference minimization (DDM) method [59] in the anisotropic approximation for all atoms. The effects of preferred orientation, anisotropic broadening of peak and sample surface roughness and displacement were taken into account during refinement. The data for the isostructural sulfides Ba_2_MnS_3_ [60], BaLaCuS_3_ [61] and KZrCuS_3_ [9] were used as initial structural models for SrLaCuS_3_, SrNdCuS_3_ and SrTmCuS_3_, respectively. The anomalous disbalance of the thermal parameters of the Sr and Ln sites after the preliminary refinement of the structures indicated a possible mixed filling of their sites due to similar ionic radii. Indeed, refinement of the occupancy of the Sr and Ln sites for the structures of SrLaCuS_3_ and SrNdCuS_3_ improved agreement between the calculated and experimental data as well as balanced the thermal parameters.

Scanning electron microscopy (SEM) was performed on a JEOLJSM-6510 LV (JEOL Ltd., Tokyo, Japan) equipped with an energy dispersive spectrometer.

The Fourier-transform infrared (FTIR) absorption spectra in the range of 60–675 cm^−1^ were recorded on a VERTEX 80v FT-IR spectrometer (Bruker OJSC, Ettlingen, Germany). The attenuated total reflectance infrared (ATR-IR) absorption spectra in the range of 400–4000 cm^−1^ were recorded on a Cary 630 FTIR spectrometer (Agilent Technologies Inc., Santa Clara, CA, USA) equipped with an ATR attachment and a DTGS detector. The Raman spectra were collected in backscattering geometry using a triple monochromator Horiba JobinYvon T64000 Raman spectrometer (Horiba Ltd., Tokyo, Japan) operating in subtractive mode. The spectral resolution for the recorded Stokes-side Raman spectra was better than 3 cm^−1^ (this resolution was achieved by using gratings with 1800 grooves mm^−1^ and 100 µm slits). The single-mode radiation at 532 nm from the Spectra-Physics Excelsior laser was used as an excitation light source, the power on the sample being 5 mW.

The diffuse reflectance spectra were recorded on a UV-2600 spectrophotometer (Shimadzu OJSC, Tokyo, Japan) equipped with an ISR-2600Plus attachment with the photomultiplier PMT of the R-928 type and InGaAs detectors. BaSO_4_ (99.8%) was used as a standard.

The temperature-dependent (20–300 K) magnetic susceptibilities of SrLnCuS_3_ (Ln = Nd, Tm) were studied on a Quantum Design MPMS3 SQUID magnetometer in a 200 Oe (15.92 kA m^−1^) magnetic field. The measurements were performed in the zero-field cooled (ZFC) and nonzero-field cooled (FC) modes. The room temperature magnetic properties of SrLnCuS_3_ (Ln = Nd, Tm) were studied on a vibrating sample magnetometer with a Puzey electromagnet [62]. The magnetic field was varied in the range –15 ÷ 15 kOe (–1.2 ÷ 1.2 MA m^−1^).

### 3.3. Synthesis

Crystalline samples of SrLnCuS_3_ (Ln = La, Nd, Tm) in the form of single crystals were obtained from a stoichiometric ratio of the elemental strontium, copper, lanthanide and sulfur in the presence of CsBr as a flux. The reaction mixture was heated in an evacuated quartz ampoule for 8 days at 1070 °C. A thin layer of graphite was deposited on the inner wall of the quartz ampoule by a pyrolytic method to avoid side reactions with quartz glass, leading to the formation of oxosilicates. The reaction product was purified from flux residues with demineralized water. The resulting yellow needle-like crystals were suitable for a single-crystal X-ray diffraction analysis.

Powdered samples of SrLnCuS_3_ (Ln = La, Nd, Tm) were prepared by reductive sulfidation of the oxide mixtures in a flow of H_2_S and CS_2_, obtained by decomposition of ammonium thiocyanate (argon was used as a carrier gas) according to a synthetic procedure reported recently [36]. According to X-ray phase analysis, the resulting samples comprised a mixture of oxides Ln_2_O_3_, SrLnCuO_4_, (Sr,Ln)_2_CuO_4_, CuSrO_2_, SrLn_2_O_4_, and Sr_x_Ln_2–x_CuO_4_, which were subjected to reductive sulfidation in a flow of H_2_S and CS_2_, yielding the titular quaternary sulfides. Sulfidation was carried out with grinding of the resulting product in three steps: heating at 870 K for 6 h, followed by heating at 1070 K for 4 h, followed by heating at 1170 K for 20 h. This stepwise increasing of the reaction temperature is of importance to avoid the decomposition of reactor material, which, in turn, leads to the formation of silicate impurities. Thus, sulfidation is initiated by isothermal heating at 870 K until the formation of sulfides and oxysulfides, which are not so aggressive with respect to the quartz material. The sulfidation product was cooled in an argon flow. The resulting products SrLnCuS_3_ were examined by SEM-EDX, and the obtained data are in agreement with the powder X-ray diffraction data and are collected in Table 6.

## 4. Conclusions

In summary, we report on a novel heterometallic quaternary sulfides SrLnCuS_3_ (Ln = La, Nd, Tm), which were synthesized both in the form of single crystals as well as powdered samples. The former synthetic procedure allows the production of pure samples but requires heating for 8 days, while the latter synthetic approach significantly decreases the reaction time to less than 2 days. However, synthesis of the powdered samples also yields some impurities, though of minor quantities. The structures of both the single crystal and powdered samples of SrLaCuS_3_ and SrNdCuS_3_ belong to the orthorhombic space group *Pnma* but of different structural types, namely Ba_2_MnS_3_, BaLaCuS_3_ and Eu_2_CuS_3_. SrTmCuS_3_ crystallizes in the orthorhombic space group *Cmcm* with the structural type KZrCuS_3_ for both the single crystal and powdered samples.

Three-dimensional crystal structures of the herein-obtained SrLaCuS_3_ and SrNdCuS_3_ are formed from the (Sr/Ln)S_7_-capped trigonal prisms as well as CuS_4_ tetrahedra, however, yielding different packing of coordination polyhedra. Particularly in SrLaCuS_3_, alternating 2D layers are stacked, while the main backbone of the structure of SrNdCuS_3_ is a polymeric 3D framework [(Sr/Ln)S_7_]_n_, strengthened by 1D polymeric chains (CuS_4_)_n_, with 1D channels, filled by the other Sr^2+^/Ln^3+^ cations, which, in turn, form 1D dimeric ribbons. A 3D crystal structure of SrTmCuS_3_ is constructed from the SrS_6_ trigonal prisms, TmS_6_ octahedra and CuS_4_ tetrahedra. The latter two polyhedra are packed together into 2D layers, which are separated by 1D chains (SrS_6_)_n_ and 1D free channels. Different crystal packings in the reported structures are, most likely, explained by both the formation of different structural types, as well as different Sr- and Ln-based coordination polyhedra. Furthermore, in both crystal structures of SrLaCuS_3_ obtained in this work, the crystallographic positions of strontium and lanthanum are partially mixed, while only in the structure of SrNdCuS_3_, solved from the powder X-ray diffraction data, are the crystallographic positions of strontium and neodymium partially mixed.

The optical properties of SrLnCuS_3_ (Ln = La, Nd, Tm) were revealed by diffuse reflectance spectroscopy, and the band gaps were found to be 1.86, 1.94 and 2.57 eV, respectively. Similar band gap values for SrLaCuS_3_ and SrNdCuS_3_ might tentatively be explained by the same orthorhombic space group *Pnma*.

Finally, SrNdCuS_3_ and SrTmCuS_3_ are paramagnetic at 20–300 K. Experimental magnetic characteristics for these sulfides are in good agreement with the corresponding calculated parameters, obtained in the model of free ions Nd^3+^ and Tm^3+^, respectively.

## Figures and Tables

**Table 1:** Experimental details for the structures of SrLnCuS_3_, solved from the single-crystal X-ray diffraction data.

	SrLaCuS_3_^a^	SrNdCuS_3_^a^	SrNdCuS_3_^a^	SrYCuS_3_^a^	SrTmCuS_3_^a^	SrScCuS_3_^a^
Space group	*Pnma*	*Pnma*	*Pnma*	*Pnma*	*Cmcm*	*Cmcm*
Structural type	Ba_2_MnS_3_	BaLaCuS_3_	Eu_2_CuS_3_	Eu_2_CuS_3_	KZrCuS_3_	KZrCuS_3_
*a* (Å)	8.1682(6)	11.0663(8)	10.5693(7)	10.1845(7)	3.9163(3)	3.8316(3)
*b* (Å)	4.0748(3)	4.0886(3)	4.0072(3)	3.9378(3)	12.9520(9)	12.8504(9)
*c* (Å)	16.0394(11)	11.4625(8)	12.8905(9)	12.9426(9)	10.0642(7)	9.7153(7)
*V* (Å^3^)	533.85(7)	518.63(2)	545.96(3)	519.06(2)	510.50(6)	478.36(2)
*Z*	4	4	4	4	4	4
*ρ* (g cm^−3^)	4.806	5.015	4.764	4.303	5.416	4.059
*μ* (mm^−1^)	22.76	25.20	23.94	26.41	32.81	18.01
Collected reflection	13821	7074	10117	8491	2756	4498
Unique reflections	846	678	738	734	340	351
*R*_int_	0.0653	0.068	0.137	0.075	0.0678	0.055
*R*_1_(all)	0.0217	0.032	0.043	0.029	0.0248	0.017
*wR*_2_(all)	0.0382	0.054	0.080	0.033	0.0426	0.032
*S*	1.022	1.059	1.004	0.941	1.007	1.031
Reference	This work	[46]	[46]	[47]	This work	[47]

^a^ Heating a stoichiometric ratio of the elements Sr, Ln, Cu and S in the presence of CsBr as a flux in an evacuated quartz ampoule for 8 days at 1070–1120 K.

**Table 2:** Experimental details for the structures of SrLnCuS_3_, solved from the powder X-ray diffraction data.

	SrLaCuS_3_^a^	SrLaCuS_3_^b^	SrCeCuS_3_^a^	SrCeCuS_3_^c^	SrPrCuS_3_^a^	SrNdCuS_3_^d^	SrSmCuS_3_^a^
Space group	*Pnma*	*Pnma*	*Pnma*	*Pnma*	*Pnma*	*Pnma*	*Pnma*
Structural type	BaLaCuS_3_	Ba_2_MnS_3_	BaLaCuS_3_	Ba_2_MnS_3_	BaLaCuS_3_	BaLaCuS_3_	Eu_2_CuS_3_
*a* (Å)	11.2415(1)	8.1746(3)	11.1626(2)	8.1393(3)	11.1171(1)	11.0815(2)	10.4285(2)
*b* (Å)	4.11053(6)	4.0727(2)	4.0970(2)	4.0587(2)	4.09492(6)	4.0849(1)	3.98640(7)
*c* (Å)	11.5990(1)	16.0473(8)	11.5307(1)	15.9661(2)	11.5069(2)	11.4684(2)	12.9325(2)
*V* (Å^3^)	535.97(1)	534.26(4)	527.33(1)	527.44(2)	523.84(1)	519.14(1)	537.63(2)
*R*_DDM_ (%)	5.25	5.73	4.52	6.61	5.03	4.00	4.94
*R*_F_ (%)	1.53	1.1	2.87	3.78	1.80	3.7	2.09
Impurity	–	2.6% SrS	–	–	–	5.2% SrS 1.3% NdCuSO	2.6% SmCuS_2_ 1.6% Sm_2_SO_2_
Reference	[43]	This work	[44]	[44]	[43]	This work	[22]
	**SrGdCuS_3_^a^**	**SrHoCuS_3_^c^**	**SrErCuS_3_^a^**	**SrErCuS_3_^c^**	**SrTmCuS_3_^e^**	**SrYbCuS_3_^c^**	**SrLuCuS_3_^a^**
Space group	*Pnma*	*Pnma*	*Cmcm*	*Cmcm*	*Cmcm*	*Cmcm*	*Cmcm*
Structural type	Eu_2_CuS_3_	Eu_2_CuS_3_	KZrCuS_3_	KZrCuS_3_	KZrCuS_3_	KZrCuS_3_	KZrCuS_3_
*a* (Å)	10.3288(2)	10.1487(1)	3.92672(5)	3.93128(3)	3.9210(1)	3.91448(4)	3.91105(4)
*b* (Å)	3.96271(7)	3.9332(1)	12.9632(2)	12.9709(1))	12.9523(5)	12.9554(1)	12.9504(1)
*c* (Å)	12.9397(2)	12.9524(2)	10.0974(1)	10.1161(1)	10.0687(4)	10.0332(1)	10.0206(1)
*V* (Å^3^)	529.62(2)	517.02(2)	513.99(1)	515.843(9)	511.34(3)	508.842(8)	507.540(8)
*R*_DDM_ (%)	4.41	4.29	5.67	3.73	4.80	3.56	5.27
*R*_F_ (%)	2.18	1.91	2.60	2.06	2.60	1.48	1.27
Impurity	3.6% GdCuS_2_ 2.5% SrS 0.8% Gd_2_SO_2_	2.6% SrS	6.3% Er_x_Cu_y_S_2_ 3.5% SrS	9.5% Er_2_SO_2_ 1.2% SrS 0.5% Er_5_S(SiO_4_)_3_	5.1% Tm_2_SO_2_ 1.2% SrS	2.2% Yb_2_SO_2_ 1.8% Yb_5_S(SiO_4_)_3_ 1.2% SrS	1.6% SrS 1.1% Lu_2_SO_2_
Reference	[22]	[36]	[22]	[45]	This work	[45]	[22]

^a^ Melting of sulfides Cu_2_S, Ln_2_S_3_ and SrS under a high-frequency current condition, followed by annealing at 970 K for 3 months. ^b^ Sulfidation of oxides (Sr_0.15_La_1.85_)CuO_4_, (Sr_0.05_La_1.95_)CuO_4_, SrLnCuO_4_ obtained by thermal decomposition of nitrates, at 1170 K. ^c^ Melting of sulfides Cu_2_S, Ln_2_S_3_ and SrS under a high-frequency current condition, followed by annealing at 1170 K for 2 months. ^d^ Sulfidation of oxides Nd_2_O_3_, Sr_2_CuO_4_ and Nd_2_CuO_4_, obtained by thermal decomposition of nitrates, at 1170 K. ^e^ Sulfidation of oxides SrTm_2_O_4_, Tm_2_O_3_, Sr_2_CuO_4_, Tm_2_CuO_4_ and CuSrO_2_, obtained by thermal decomposition of nitrates, at 1170 K.

**Table 3:** Fractional atomic coordinates and occupancy of SrLnCuS_3_ (Ln = La, Nd, Tm).

Atom	*x*	*y*	*z*	Occupancy	*x*	*y*	*z*	Occupancy
	SrLaCuS_3_ (single crystal)				SrLaCuS_3_ (powdered sample)			
Sr1	0.09058(3)	1/4	0.785319(17)	0.502(5)	0.09026(6)	1/4	0.785177(28)	0.5453(46)
La1	0.09058(3)	1/4	0.785319(17)	0.498(5)	0.09026(6)	1/4	0.785177(28)	0.4547(46)
Sr2	0.25439(3)	1/4	0.038345(17)	0.489(6)	0.25446(6)	1/4	0.038210(26)	0.4550(43)
La2	0.25439(3)	1/4	0.038345(17)	0.511(6)	0.25446(6)	1/4	0.038210(26)	0.5450(43)
Cu	0.11864(6)	1/4	0.36655(3)	1	0.11857(11)	1/4	0.36652(6)	1
S1	0.17928(12)	1/4	0.22120(6)	1	0.17956(18)	1/4	0.22114(9)	1
S2	0.38083(12)	1/4	0.42864(6)	1	0.38097(19)	1/4	0.42837(10)	1
S3	0.01190(12)	1/4	0.60039(6)	1	0.01197(18)	1/4	0.60006(10)	1
	SrNdCuS_3_ (single crystal) [46]				SrNdCuS_3_ (powdered sample)			
Sr1	0.31732(6)	1/4	0.49523(6)	1	0.31752(15)	1/4	0.49500(17)	0.886(5)
Nd1	0.48946(3)	1/4	0.81684(4)	1	0.31752(15)	1/4	0.49500(17)	0.114(5)
Sr2	–	–	–	–	0.48947(13)	1/4	0.81683(11)	0.114(5)
Nd2	–	–	–	–	0.48947(13)	1/4	0.81683(11)	0.886(5)
Cu	0.24480(8)	1/4	0.21334(9)	1	0.2447(2)	1/4	0.2133(3)	1
S1	0.22363(17)	1/4	0.80669(16)	1	0.2250(5)	1/4	0.8076(4)	1
S2	0.04818(16)	1/4	0.14176(17)	1	0.0487(4)	1/4	0.1406(4)	1
S3	0.38733(17)	1/4	0.05848(17)	1	0.3872(4)	1/4	0.0571(4)	1
	SrTmCuS_3_ (single crystal)				SrTmCuS_3_ (powdered sample)			
Sr	0	0.74817(7)	1/4	1	0	0.74800(12)	1/4	1
Tm	0	0	0	1	0	0	0	1
Cu	0	0.47124(9)	1/4	1	0	0.47147(17)	1/4	1
S1	0	0.36340(13)	0.06401(14)	1	0	0.3634(2)	0.0644(2)	1
S2	0	0.07621(18)	1/4	1	0	0.0761(3)	1/4	1

**Table 4:** Bond lengths (Å) in the crystal structures of SrLnCuS_3_ (Ln = La, Nd, Tm). For symmetry codes see Figure 1.

SrLaCuS_3_ (Single Crystal/Powdered Sample)					
Sr1/La1–S1	2.957(1)/2.9569(11)	Sr2/La2–S1	2.996(1)/2.9988(16)	Cu–S1	2.383(1)/2.3857(18)
Sr1/La1–S1^i^	2.957(1)/2.9569(11)	Sr2/La2–S2^iv^	2.910(1)/2.9119(12)	Cu–S2^x^	2.362(1)/2.3636(18)
Sr1/La1–S1^ii^	3.003(1)/3.0036(11)	Sr2/La2–S2^v^	2.910(1)/2.9119(12)	Cu–S3^vi^	2.360(1)/2.3607(9)
Sr1/La1–S1^iii^	3.003(1)/3.0036(11)	Sr2/La2–S2^vi^	3.097(1)/3.0999(17)	Cu–S3^ix^	2.360(1)/2.3607(9)
Sr1/La1–S2	3.081(1)/3.0793(13)	Sr2/La2–S3	2.964(1)/2.9627(11)		
Sr1/La1–S2^i^	3.081(1)/3.0793(13)	Sr2/La2–S3^vii^	2.964(1)/2.9627(11)		
Sr1/La1–S3	3.035(1)/3.0387(16)	Sr2/La2–S3^viii^	3.062(1)/3.0585(16)		
**SrNdCuS_3_ (Single Crystal/Powdered Sample)**					
Sr1/Nd1–S1	3.009(2)/3.002(4)	Sr2/Nd2–S1	2.944(2)/2.933(5)	Cu–S1	2.334(1)/2.335(3)
Sr1/Nd1–S1^i^	3.009(2)/3.002(4)	Sr2/Nd2–S1^v^	2.953(2)/2.974(5)	Cu–S1^i^	2.334(1)/2.335(3)
Sr1/Nd1–S2^ii^	3.036(2)/3.026(4)	Sr2/Nd2–S2	2.895(1)/2.904(4)	Cu–S2	2.325(2)/2.327(5)
Sr1/Nd1–S2^iii^	3.036(2)/3.026(4)	Sr2/Nd2–S2^vi^	2.895(1)/2.904(4)	Cu–S3	2.375(2)/2.388(5)
Sr1/Nd1–S2^iv^	2.999(2)/2.997(5)	Sr2/Nd2–S3^ii^	2.992(2)/2.980(5)		
Sr1/Nd1–S3^ii^	3.136(2)/3.135(4)	Sr2/Nd2–S3^v^	2.843(1)/2.851(3)		
Sr1/Nd1–S3^iii^	3.136(2)/3.135(4)	Sr2/Nd2–S3^vii^	2.843(1)/2.851(3)		
**SrTmCuS_3_ (Single Crystal/Powdered Sample)**					
Sr–S1^ii^	3.093(1)/3.093(2)	Tm–S1	2.717(1)/2.719(2)	Cu–S1	2.336(2)/2.335(3)
Sr–S1^v^	3.093(1)/3.093(2)	Tm–S1^i^	2.717(1)/2.719(2)	Cu–S1^viii^	2.336(2)/2.335(3)
Sr–S1^vi^	3.093(1)/3.093(2)	Tm–S1^iii^	2.717(1)/2.719(2)	Cu–S2	2.384(2)/2.383(3)
Sr–S1^vii^	3.093(1)/3.093(2)	Tm–S1^iv^	2.717(1)/2.719(2)	Cu–S2^i^	2.384(2)/2.383(3)
Sr–S2	2.966(2)/2.966(3)	Tm–S2	2.703(1)/2.703(2)		
Sr–S2^i^	2.966(2)/2.966(3)	Tm–S2^ii^	2.703(1)/2.703(2)		

**Table 5:** Magnetic characteristics for SrNdCuS_3_ and SrTmCuS_3_.

	SrNdCuS_3_	SrTmCuS_3_
Space group	*Pnma*	*Cmcm*
Structural type	Ba_2_MnS_3_	KZrCuS3
Calculated *μ* (μ_B_)	3.618	7.561
Experimental *μ*_296 K_ (μ_B_)	3.611	7.378
Experimental *μ*_20–300 K_ (μ_B_)	3.52	7.57
Calculated *C* (K m^3^ kmol^−1^)	0.02057	0.08983
Experimental *C*_20–300 K_ (K m^3^ kmol^−1^)	0.0195	0.0901
Experimental *θ*_20–300 K_ (K)	−18	−12

**Table 6:** The calculated and found elemental analysis data for SrLnCuS_3_ (Ln = La, Nd, Tm) obtained using SEM-EDX.

Compound (Mass)	Calculated (%)					Found (%)				
	Sr	Ln	Cu	S	O	Sr	Ln	Cu	S	O
SrLaCuS_3_ (386.25)	22.68	35.96	16.45	24.90	–	23.23	35.82	16.06	24.89	–
97.4% SrLaCuS_3_ + 2.6% SrS	23.10	35.67	16.32	24.92	–					
SrNdCuS_3_ (391.59)	22.38	36.83	16.23	24.56	–	24.68	35.18	15.52	24.54	0.08
93.5% SrNdCuS_3_ + 5.2% SrS + 1.3% NdCuSO	23.02	36.40	16.04	24.49	0.06					
SrTmCuS_3_ (416.28)	21.05	40.58	15.27	23.10	–	20.53	42.51	14.25	22.30	0.41
93.7% SrTmCuS_3_ + 1.2% SrS + 5.1% Tm_2_SO_2_	20.18	42.60	14.45	22.36	0.40					
